# The Disorders of Primary Dentition

**Published:** 1882-08

**Authors:** M. Blanches


### ARTICLE VII.

### *The Disorders of Primary Dentition.*—(Continued.)

If these facts are not constantly observed, they are at least common, and it is certainly very rare to find a child who has not suffered more or less during the period of dentition. I have observed, for my part, a child who never had one of his first twenty teeth without suffering from one or several convulsions, and who has never presented any since the period of dentition.

What we have said of the fever, diarrhœa, and nervous disorders, can be repeated with equal justice for the multiform eruptions frequently observed in similar cases. There is nothing special to distinguish these eruptions, any more than the intestinal or nervous disorders, from those produced by other and widely different causes. They demonstrate, however, the existence of disorders in the functions of the various organs, and in the secretions, produced by dentition.

What especially characterizes these eruptions is the period of their appearance and the manner in which they follow "the evolution of the teeth, preceding and accompanying the irruption of a group of teeth and then disappearing."

If all the morbid symptoms observed during dentition cannot be ascribed to this physiological act, it is not the less true that these disorders, appearing only at this period, accompanying the irruption of the teeth and ceasing when this is accomplished, do not leave in doubt their veritable causation.

It may be admitted that dentition is not an isolated phenomenon; that it coincides with a period of peculiar activity in the development of the child, principally with the formation of the bones, the increase of the glandular system of the digestive apparatus, and at this moment the nervous sensibility is at its highest pitch.

This is all true, but it is certain that dentition, particularly when painful, has a preponderating role in the causation of these morbid manifestations.

After denying that teething can be of itself painful, M. Magitot demonstrates that traumatisms practised on young animals at the period of dentition, and affecting more or less deeply the gum or the tooth, do not determine anything comparable to the disorders attributed in children to the evolution of the teeth.

We are of opinion that there can be no analogy between the sections, punctures and tearing of the alveoli in these experiments, and the normal evolution of the tooth, which separates, forces out and slowly compresses the tissues. This last is a purely vital act, not in any manner reproduced in the traumatisms already mentioned, which induce widely different reactions.

In the same way the numerous diseases of the teeth, caries, ulcerations, etc., may often induce very severe pain without provoking any symptom resembling those observed during the period of the evolution of the teeth. The reactions are peculiar to this period and are analogous to those observed during the evolution of other organs.

The opinions of M. Magitot are reproduced in the ably and conscientious thesis presented by M. Leveque to the Paris Faculty.

We have particularly considered the seven observations given by M. Leveque, and we find that several might be given as types of the disorders induced by dentition, and the interpretation of them given by the author is, to say the least, extremely far-fetched. But these objections do not mar the value of the work; it is possible that the author will persist in his ideas if he practices dentistry exclusively. But it would be entirely different if he is called upon to follow, in their maladies and indispositions, young infants, to have them under his care during their development, and be obliged to interpret their sufferings. He would then remark, with all clinical observers, that dentition exercises a very marked influence in the production of infantile disorders, and he would soon recognize how solidly the opinion he combats to-day is established.—*M. Blanchez, in Med. and Surg. Reporter.*

---

